# Extent of biodiversity surveys and ranges for endemic species in the Albertine Rift

**DOI:** 10.1016/j.dib.2018.04.111

**Published:** 2018-05-04

**Authors:** S. Ayebare, A.J. Plumptre, D. Kujirakwinja, D. Segan

**Affiliations:** a Wildlife Conservation Society, 2300 Southern Boulevard, Bronx, NY 10460, USA; b Conservation Science Group, Department of Zoology, Pembroke Rd, Cambridge, UK

**Keywords:** Biodiversity surveys, Albertine Rift, Endemic species ranges, Climate change, Habitat loss

## Abstract

This article contains data on the estimated ranges of endemic species in the Albertine Rift both currently and under future climate change related to the research article entitled “Conservation of the endemic species of the Albertine Rift under future climate change” (Ayebare et al., 2018) [1]. Biodiversity surveys focused mainly on 5 taxa: birds, mammals, reptiles, amphibians and plants. A combination of line transects, point counts, recce walks, camera traps, visual encounter surveys, qualitative surveys and appropriate capture methods (mist nets, Sherman traps, pitfall traps) were used to survey the different taxa and provide point location data for each species. The biodiversity surveys were conducted by the Wildlife Conservation Society starting in the late 1990s. Additional species data were sourced from individual researchers and institutions.

The current and future species ranges were estimated using the Maximum Entropy ‘(Maxent)’ species distribution modeling algorithm. The areas of suitable habitat (current and future) of 162 endemic species for 5 taxa (birds (40), mammals (33), plants (49), reptiles (11), amphibians (29), the extent of occurrence (EOO), area of occupancy (AOO), percentage range contraction due to climate change and to agriculture conversion of suitable habitat for each species are given in Table S1. Threshold geotiffs are also provided for each species modeled and made available at www.albertinerift.org.

**Specifications Table**

**Table:** 

Subject area	Conservation Biogeography
More specific subject area	Albertine Rift; Endemic species ranges
Type of data	Tables, Excel files, Figures, Raster geotiffs
How data was acquired	Field surveys
Data format	Analysed
Experimental factors	Not applicable
Experimental features	Not applicable
Data source location	Kampala, Uganda, Wildlife Conservation Society
Data accessibility	Some of the data are with this article. Additional data is available on the WCS web site www.albertinerift.org

**Value of the data**

• Mapping the biodiversity patterns of vertebrates and plants across the Albertine Rift.
• Assessment of loss of suitable habitat to agriculture for each species.
• Assessing species vulnerability to changes in climate under different greenhouse gas emission scenarios and General Circulation models.
• Assessing species threat status under the IUCN Red List criteria predicting loss under future climate change.
• Conservation planning under climate change and other competing land uses.

## 1 Data

Suitable habitat for the endemic species of the Albertine Rift region (current and future ranges) were estimated using the Maximum Entropy (Maxent), species distribution modeling algorithm [1], [2]. Climate predictor variables used in the species distribution modeling were downloaded from the WorldClim database (Table 1; [1], [3]). The projected future ranges were based on the 2080 estimates from the Special Report on Emissions Scenarios (SRES) and 2070 for the Representative Concentration Pathways (RCPs) (Table 2).

**Table 1:** Covariates used for modelling the distribution of endemic species in the Albertine Rift.

**Covariate**	**Description of variable**	**Reference**
Bio2	Mean daily temperature range	[3]
Bio7	Temperature annual range	[3]
Bio6	Minimum temperature of coldest month	[3]
Bio5	Maximum temperature of warmest month	[3]
Bio12	Annual precipitation	[3]
Bio17	Precipitation of driest quarter	[3]
Bio16	Precipitation of wettest quarter	[3]

**Table 2:** Temperature projections for the four Representative Concentration Pathways (CMIP5) and how they relate to the Special Report on Emissions Scenarios (CMIP3).

RCPs/CMIP5	Median (°C)	Description	SRES/CMIP3	Median (°C)
RCP2.6		Radiative forcing peak at approximately 3 W/m^2^ before 2100 and then decline		
RCP4.5,	2.4	This is an intermediate stabilization pathway in which the radiative forcing is stabilized at approximately 4.5 W/m^2^ after 2100	SRES B1	2.3
RCP6	2.9	This is an intermediate stabilization pathway in which the radiative forcing is stabilized at approximately 6 W/m^2^ after 2100	SRES B2	2.9
RCP8.5:	4.6	This is the highest concentration pathway in which the radiative forcing reaches > 8.5 W/m^2^ by 2100 and continues to rise	SRES AIF1	4.5
			SRES A2	3.9

**Fig. 1:**
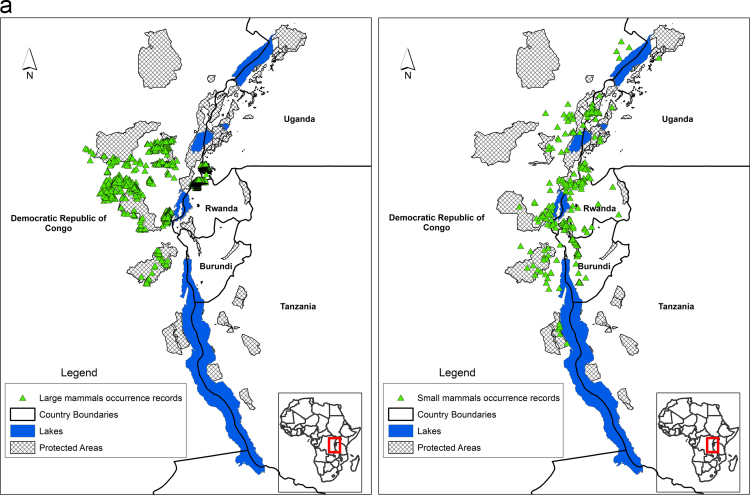

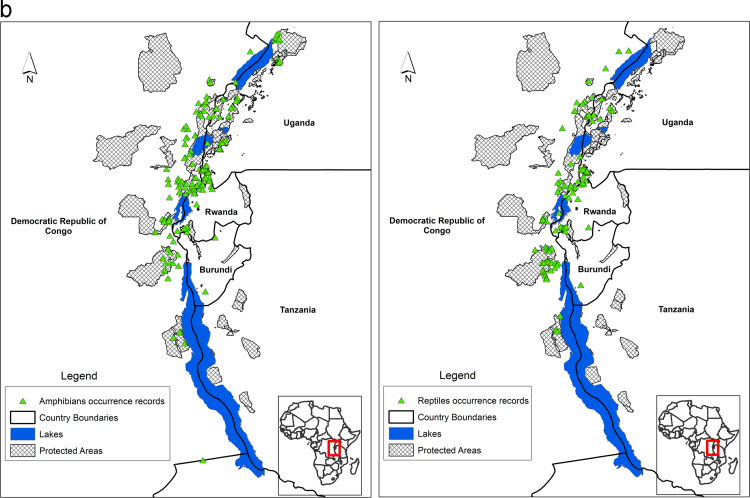

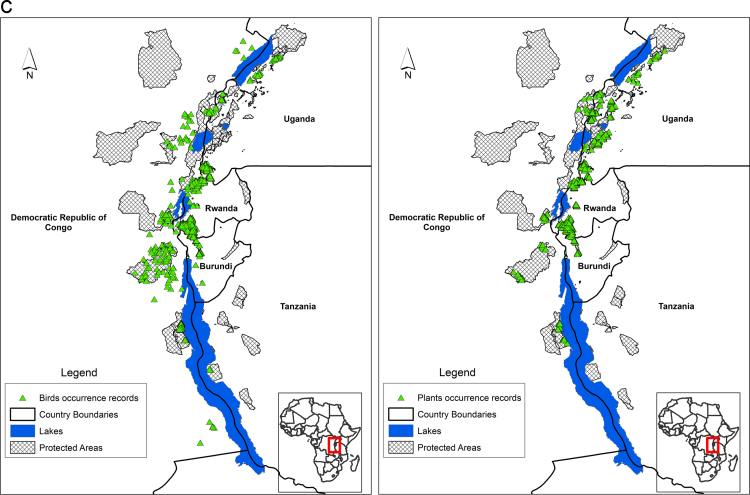
**a** Species occurrence records for large mammals (left) and small mammals (right). **b.** Species occurrence records for amphibians (left) and reptiles (right).**c.** Species occurrence records for birds (left) and plants (right).

Species' occurrence records from biodiversity surveys for large mammals, small mammals, amphibians, reptiles, birds and plants is mapped across the Albertine Rift (Fig. 1a, 1b, 1c). These data were collected by Wildlife Conservation Society biodiversity survey team since the early 1990s. Additional data for western Tanzanian large mammals was obtained from the Tanzanian mammal atlas (courtesy of Charles Foley), Global Biodiversity Information Facility [4], records of amphibian collections made by Mathias Behangana (Makerere University), Michele Menegon (Trento Science Museum) and Eli Greenbaum (University of Texas at el Paso), and small mammal collections by Julian Kerbis Peterhans (Chicago Field Museum). Areas of each species’ range lost to agriculture were estimated by clipping predicted suitable habitat from the current Maxent threshold raster by an agriculture layer for the region that mapped presence/absence of agriculture (see below). Area lost to climate change was estimated for each species by measuring the areas of the threshold suitability maps for the current maps and the maps from 2070. These results are given for each species in Table S1 together with areas of overlap between current and future ranges of each species.

The climate data used for the estimation of future ranges were from the Coupled Model Inter-comparison Project Phase3 (CMIP3) but more recent global climate models have been developed under the framework of the Coupled Model Inter-comparison Project Phase5 (CMIP5). It was not possible to remodel all of the species but to assess the effects of the newer models, we compared 20 of the endemic birds modeled using both pathways to assess what differences exist as a result of the newer models (Table S2).

## 2 Experimental design, materials and methods

**Fig. 2:**
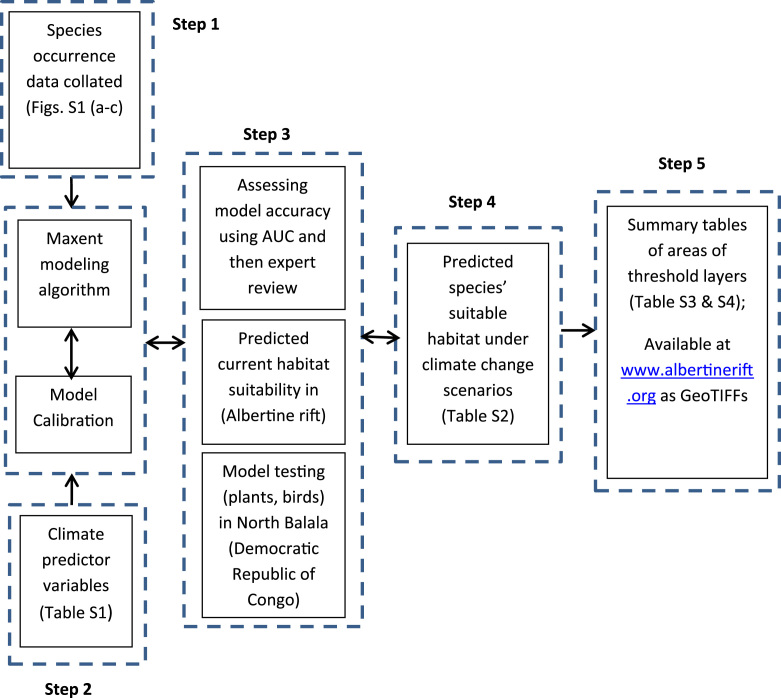
Schematic showing the steps used in producing species threshold layers from niche modeling (modified from Peterson et al. [9]).

A conceptual framework for the analysis is given in Fig. 2. A combination of line transects, point counts, recce walks, camera traps and appropriate capture methods were used to survey the different taxa across the Albertine Rift by the Wildlife Conservation Society and its partners.

### 2.1 Bird surveys

The point count method was used to survey birds. All birds seen or heard were recorded by the observers at 250 m intervals along transects/recces. The observers waited a couple of minutes for birds to adjust to their presence and then made five minute point counts. Birds were identified visually or by their calls at every point visited and a GPS reading was taken. Two observers would stand at every point and record all the birds in the following distance classes: 0–10 m, 10–20 m, 20–50 m, 50–100 m, 100–200 m, 200–500 m for five minutes.

### 2.2 Mammal surveys

Line transect sampling methods [5] and recce walks were used to survey large mammals. The number of animals seen along transects/recce walks and indirect signs of species’ presence were recorded. In some protected areas camera traps were installed to observe cryptic or elusive animals. Capture methods were used to survey small mammals (bats, rodents and shrews). Mist netting was used to survey bats at various sites. Sherman traps were set along line transects and baited with peanut butter, maize flour, ghee or fish.

### 2.3 Amphibians and reptiles (herpetofauna)

Visual encounter surveys (VES) and pitfall trapping were used for conducting surveys of herpetofauna. During the VES method, the observer moves through the habitat watching out for and recording surface-active species. Pitfall traps were set up with a drift fence to sample any surface dwelling herpetofauna.

### 2.4 Plant surveys

Standard nested circular plots located at 250 m intervals along transects were used in the survey of plants. The circular plots consist of a 20 m radius plot (trees greater than 10 cm DBH (Diameter at breast height) are identified and counted), 10 m radius plot (lianas, shrubs and trees less than 10 cm DBH but greater than 2.5 cm DBH are identified and counted) and a 2 m radius plot (all grasses and herbs are identified). Sample specimens were collected, dried and pressed on site for all uncertain species, to enable confirmation of identification at a Herbarium.

### 2.5 Species modeling

Details on the modeling are given in [1] and are summarised in Fig. 2. We selected 17 Worldclim variables relating to climate that are likely to influence the distribution of the birds, mammals, plants, reptiles and amphibians in the Albertine Rift. All the predictor variables were clipped to the area of interest and a pairwise Pearson correlation between predictor variables were obtained using ENMTOOLs [6]. To minimize the effect of multi-collinearity and overfitting, only seven variables with less than (± 0.75) correlation were retained (Table 1). Threshold layers, representing binary (presence/absence) predictions of the distribution for each species were made maximising sensitivity and specificity [7].

### 2.6 Mapping agriculture

To remove suitable habitat lost to agriculture from each species range the GlobCover 2009 land cover map was re-classified into three land cover classes; water, other, and natural vegetation [8]. The “other” class was characterized by agriculture areas, plantation forestry and human settlements. When exploring the GlobCover 2009 land cover map in ArcGis 9.3 we found that there were vegetation misclassifications in our study area. For example some of the high tropical forest reserves had been classified as mosaic vegetation/cropland. We edited the GlobCover 2009 landcover map by locking in areas we knew to be natural vegetation and using other landcover maps for the region to produce a final layer used for removing modified habitat from the species range maps. This map was then used to clip the current threshold maps to estimate the percentage of suitable habitat lost to agriculture for a species.

## Appendix A Supplementary material

Supplementary material Supplementary data associated with this article is contained in excel files (Tables S1 and S2) and can be found in the online version at. The estimated ranges in raster format are available at www.albertinerift.org. Table S1. Excel file of current range size, area cultivated, predicted range contractions under climate change , current and future area protected and percentage overlaps for each species assessed.

.

Supplementary material Supplementary data associated with this article is contained in excel files (Tables S1 and S2) and can be found in the online version at. The estimated ranges in raster format are available at www.albertinerift.org. Table S2. Excel file comparing the current range size, area cultivated, predicted range contractions under climate change for 20 bird species for CMIP3 and CMIP5.
