# Supplementary material for: Extent of biodiversity surveys and ranges for endemic species in the Albertine Rift
Source: Data Brief. 2018 May 4;18:1907–13. doi: 10.1016/j.dib.2018.04.111 (PMC5998652; doi:10.1016/j.dib.2018.04.111)
Supplement: Supplementary file 1 — Transparency document [file mmc1.docx]

**Conflict of Interest**

We would like to confirm that there are no conflicts of interest associated with this publication.

Declarations of interest: none
